# The Rate of Insulin use and Suboptimal Glycemic Control among Egyptian Patients with T2DM: Cohort Analysis of Eighth Wave of the International Diabetes Management Practices Study (IDMPS)

**DOI:** 10.2174/1573399820666230602100629

**Published:** 2024-01-30

**Authors:** Samir Helmy Assaad Khalil, Mohsen Khaled, Raafat Zakhary, Mark Shereen

**Affiliations:** 1 Department of Internal Medicine, Unit of Diabetology, Lipidology & Metabolism, Alexandria Faculty of Medicine, Alexandria University, Alexandria, Egypt;; 2 Diabetes and Endocrinology Department, National Institute of Diabetes and Endocrinology, Cairo, Egypt;; 3 Consultant of Diabetes and Internal Medicine, Alexandria University, Alexandria, Egypt;; 4 Department of Medical Affairs, Sanofi, Cairo, Egypt

**Keywords:** Insulin therapy, diabetes mellitus, type 2 diabetes, suboptimal glycemic, IDMPS wave 8, Egyptian patients

## Abstract

**Aims:**

The International Diabetes Management Practices Study (IDMPS) is an international annual survey aiming to study and characterize the current standards of care for managing DM in developing countries.

**Background:**

In Egypt, DM represents a substantial burden on the healthcare system, with an estimated 10.9 million patients, ranking it 10^th^ amongst countries with the highest prevalence of DM. Previous studies showed that to maintain safety and achieve treatment goals among diabetic patients, optimal insulin therapy should be selected individually based on the patient’s needs. We reported the proportion of Egyptian T2DM patients on insulin therapy who participated in the eighth wave of the IDMPS.

**Methods:**

The 2018 IDMPS wave consisted of cross-sectional and longitudinal phases and aimed to evaluate the proportion of T2DM who were on insulin therapy in 13 countries from four regions. In Egypt, 17 physicians agreed to participate in the present study and were required to include at least one patient.

**Results:**

A total of 180 T2DM patients were included in the cross-section phase. At the end of the ninth month of follow-up, data from 170 T2DM patients were available. A total of 39 T2DM patients (21.7%) were on insulin therapy, with a mean duration of 32.4 ± 36.6 months. More than half of the patients (n = 22; 56.4%) were on basal insulin, mainly long-acting (n = 20; 90.9%). The mean basal insulin daily dose was 0.3 ± 0.1 IU/Kg. Notably, 28.2% of the patients received insulin *via* vials, and 46.2% stated that they were adjusting the insulin dose by themselves. On the other hand, 60.2% of the study population was on oral antidiabetic drugs at the cross-sectional phase. Nearly 17.4% and 27% of the patients in the cross-sectional phase achieved the glycemic target per recommendations of international guidelines and the treating physicians, respectively. At the end of the longitudinal phase, the percentage of T2DM patients who achieved glycemic targets increased to 38.4% and 77.4% as per recommendations of international guidelines and the treating physicians, respectively. Overall, 38.3% of T2DM patients received diabetes education, and 28.9% were involved in an educational program provided by the physician or their clinical staff. Besides, 85.5% of T2DM patients followed their diabetes medication dosage and frequency strictly as prescribed.

**Conclusion:**

The proportion of insulin use in patients with T2DM aligned with the previous studies from different countries; however, it is still inadequate to achieve the targeted glycemic control. Nearly one-third of Egyptian patients received diabetes education, highlighting the need for adopting a national educational program. Nonetheless, the level of adherence among T2DM from Egypt appears to be high.

## 1 INTRODUCTION

Type 2 diabetes mellitus (T2DM) is a progressive condition that results from insulin resistance and progressive impairment of insulin secretion [1]. T2DM is the most common type of diabetes, accounting for over 90% of all patients with diabetes mellitus (DM) worldwide [2]. According to the 10^th^ edition of the International Diabetes Federation (IDF) diabetes atlas, up to 537 million people live with diabetes worldwide, which is expected to reach 783 million by 2045. In Egypt, DM represents a substantial burden on the healthcare system, with an estimated 10.9 million patients, ranking it 10^th^ among countries with the highest prevalence of DM [3].

Early in disease progression, oral hypoglycemic agents (OHAs) effectively lower insulin resistance or improve insulin secretion [4, 5]. However, many clinical trials showed that hemoglobin A1c (HbA1c) levels were frequently reduced by less than 1% in individuals on OHAs [6]. On the other hand, insulin is found to be effective in all stages, especially when the optimal OHAs fail to achieve glycemic control. To maintain safety and achieve treatment goals, optimal insulin therapy should be selected individually based on the patient’s needs [1]. Insulin treatment lowers microvascular complications, according to a number of major randomized clinical trials [7, 8]. Furthermore, the UK Prospective Diabetes Study (UKPDS) demonstrated that early insulin therapy in T2DM patients reduces macrovascular complications [9].

Nevertheless, the associated risk of hypoglycemia episodes limits the flexibility of insulin titration in clinical settings, especially when intensive insulin therapy is necessary [10]. Hypoglycemia is a predictor of poor outcomes in patients with T2DM, and it elevates the risk of early mortality [11, 12]. As a result, most clinical practice guidelines emphasize that adequate glycemic control in T2DM patients should be accomplished while minimizing the risk of hypoglycemia [13]. To achieve this, the physician should consider the cost, adherence, glycemic control, adverse events, and quality of life while choosing a therapy.

Generally, once-daily basal insulin should be added to oral antidiabetic drugs (OADs) at the start of insulin treatment in T2DM [1]. Furthermore, insulin should be considered at any stage if HbA1c is very high (> 11%) and/or there are symptoms or evidence of catabolism [14]. Several studies have shown that early initiation of a basal insulin regimen, followed by the addition and escalation of prandial or premixed insulin in addition to basal insulin, and finally a complete basal-bolus regime, may be the best insulin treatment for T2DM [15, 16]. Despite the promising role of insulin in controlling T2DM, the use of insulin therapy in such cases is still lower than recommended.

The International Diabetes Management Practices Study (IDMPS) is an international annual survey that aims to study and characterize the current standards of care for managing DM in developing countries. The eighth wave of the IDMPS included 13 countries from four regions (the Middle East and Africa, Europe, Latin America, and Asia). It evaluated the global proportion of patients with T2DM treated with insulin and the care management of T2DM patients in routine medical practice. In this study, we reported the results of the eighth wave of the IDMPS from Egypt.

## 2 METHODS

The present study gained the ethical approval of the local ethics committee (IRB No: 00007555-FWA No: 00018699). We confirmed that all study procedures adhere to the principles of the latest version of the Declaration of Helsinki [17] and applicable local regulatory laws. Eligible patients were required to sign the informed consent before the study’s enrolment.

### 2.1 Study Design and Setting

The IDMPS is an international observational study that assesses the current standards of care for managing T1DM and T2DM patients. The IDMPS is composed of consecutive waves; each of them aims to evaluate the current landscape of various aspects of managing DM in developing countries across the globe. The first wave of the IDMPS started in 2005 and was followed by another six waves that ended in 2017. The 2018 wave consisted of cross-sectional and longitudinal phases and aimed to evaluate the proportion of T2DM who were on insulin therapy in 13 countries from four regions (the Middle East and Africa, Europe, Latin America, and Asia), as well as the management of care of T1DM and T2DM patients in these countries. The cross-sectional phase recruited 243 physicians (76.1% diabetes specialists) and 3583 patients over a two-week enrolment period. Of them, 3162 patients were followed up for nine months to complete the longitudinal phase of the study.

In Egypt, 17 physicians (six specialists and 11 non-specialists) agreed to participate in the present study and were required to include at least one patient. It was assumed that each physician would enroll at least 10 T2DM patients visiting them during the two-week recruitment period of the cross-sectional phase of the study. The mean age of all physicians was 56.4 ± 10.3 years, with 94.1% of males. The participating physicians had been practicing medicine for 39.7 ± 4.2 years on average for specialists and 27.4 ± 10.2 years for non-specialists. The large majority of the physicians (94.1%) declared that they follow clinical practice guidelines, mainly the American Diabetes Association (ADA) or European Association for the Study of Diabetes (EASD) (87.5%). Regarding managing diabetic patients, on average, specialists reported that they usually saw 21.8 patients per day and non-specialists, 22.3 patients per day.

### 2.2 Eligibility Criteria of the Study Participants

Patients were deemed eligible if they were older than 18 years old, had an established diagnosis of T2DM according to the ADA criteria, and were visiting their treating physician during the two-week enrolment period of the cross-sectional phase of the study [18]. We excluded patients enrolled in clinical trials at the time of enrolment and/or with a history of enrolment in a previous wave of IDMPS.

### 2.3 Variables and Data Collection Methods

Data were collected through a standardized Case Report Form (CRF) for each eligible patient, which was fulfilled by the recruiting physician. At the cross-sectional phase of the study, the collected data included demographic and socio-economic characteristics of the patients, diabetes-related history, relevant medical history, anthropometric measures of the patients, blood pressure measures, glycemic parameters (HbA1c, fasting [FBG], and postprandial blood glucose [PPBG]), lipid profile, kidney function tests, the pattern of prescription of OADs and glucagon-like peptide 1 (GLP-1) receptor agonist, pattern and utilization of insulin therapy, patient education, and adherence to medications. At the end of the longitudinal phase of the study, patients were assessed for changes in glycemic parameters, achievement of treatment targets, the pattern of medication prescription, and medication adherence.

### 2.4 Study Outcomes

The primary outcome of the present study was to estimate the proportion of patients with T2DM treated with insulin in Egypt. At the same time, the secondary outcomes included the management of care of patients with T2DM in current medical practice at the cross-sectional phase, the change in glycemic parameters at the end of the longitudinal phase, and the change in the prescription pattern of antidiabetic medications, and level of adherence to the T2DM treatment.

### 2.5 Statistical Methods and Sample Size

The sample size was determined on a country basis, based on the primary objective to estimate the proportion of patients with T2DM treated with insulin overall in the whole population of the cross-sectional phase. Based on the assumption that the proportion of patients with T2DM treated with insulin was around 40%, a precision of at least 2%, and considering each physician was to enroll 10 patients with T2DM, in Egypt, it was planned to select 18 physicians and recruit 180 T2DM patients.

The quantitative variables were summarized using the mean and standard deviation (SD). The qualitative variables were summarized using the count and percentages. The statistical analyses were conducted with the SAS Software version 9.2.

## 3 RESULTS

A total of 180 T2DM patients were included in the cross-section phase. At the end of the ninth month of follow-up, data from 170 T2DM patients were available.

### 3.1 Demographic and Clinical Characteristics

The mean age of the patients in the cross-sectional phase was 53.6 ± 11 years old. Nearly 46% of the patients were males, and over 90% were from urban areas. Almost 52% of the patients had no health insurance. The mean body mass index (BMI) was 31.07 ± 5.81 kg/m^2^. Besides, 47.1% of the patients were hypertensive. Of them, 93.8% were on antihypertensive medications. Overall, 26.1% of the T2DM patients had dyslipidemia. On the other hand, two T2DM patients (2%) had stage 4-5 chronic kidney disease (CKD). Concerning DM characteristics, the mean disease duration was 8.8 ± 7.4 years for T2DM patients. Nearly 82.6% of T2DM patients had poor glycemic control (defined as HbA1c ≥7%) (Table **1**).

### 3.2 The Proportion of T2DM Patients Treated with Insulin at Cross-sectional and Longitudinal Phases

**Fig. (1):**
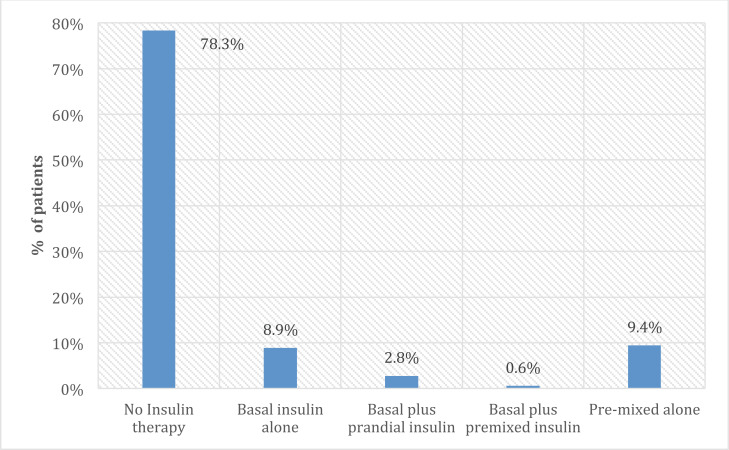
The proportion of T2DM on insulin therapy.

A total of 39 T2DM patients (21.7%) were on insulin therapy (Fig. **1**), with a mean duration of 32.4 ± 36.6 months; of them, 28 (75.7%) patients started insulin therapy for at least three months before the study’s initiation. The insulin was mainly received with OADs (n =29; 74.4%). More than half of the patients (n= 22; 56.4%) were on basal insulin, mainly long-acting (n = 20; 90.9%). Out of the 22 patients on basal insulin, 16 patients (72.7%) were on basal insulin alone, five patients (22.7%) were on basal plus prandial insulin, and one patient received basal plus premixed insulin. The mean basal insulin daily dose was 0.3 ± 0.1 IU/Kg. A total of 18 (46.2%) patients were on premixed insulin, with human-premixed insulin accounting for the vast majority of these cases (83.3%). Five (12.8%) patients were on prandial insulin (four patients on rapid-acting insulin analogues) combined with basal analogues, with a mean daily dose of 0.2 IU/Kg. Notably, 28.2% of the patients received insulin *via* vials, and 46.2% stated that they were adjusting the insulin dose by themselves (Table **2**).

All patients treated with insulin at baseline were treated with insulin at the end of follow-up. Changes in insulin treatment during the longitudinal period were reported in one patient. None of the patients discontinued insulin during the longitudinal period.

### 3.3 Management of Care of T2DM Patients

#### 3.3.1 Glycemic Control at the Cross-sectional and Longitudinal Phases

At the cross-sectional phase of the study, 17.4% and 27% of the T2DM patients achieved the glycemic target per recommendations of international guidelines (HbA1c < 7%) and treating physicians, respectively. In the subgroup of patients with suboptimal glycemic control, the main reasons for not achieving glycemic goals were the lack of diabetes education (52.8%), the lack of support (39.3%), and day-to-day blood glucose level instability (34.8%). None of the T2DM patients reached the triple target (defined as HbA1c <7%, and normal blood pressure [SBP/DBP: 130/80 mmHg] and LDL‐CS <100 mg/dL as per recommendations of international guidelines).

**Fig. (2):**
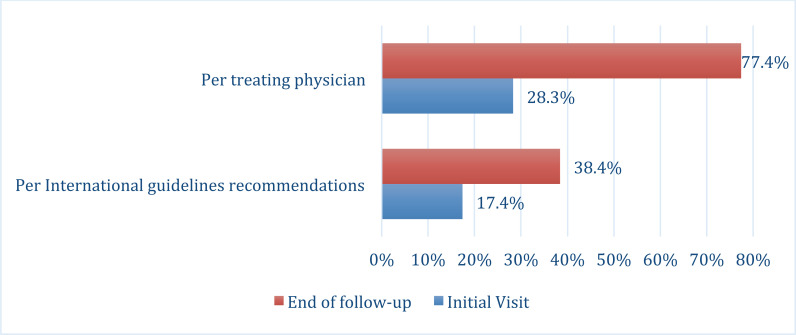
The distribution of glycemic targets at the initial and end of follow-up visits.

A total of 170 T2DM patients had available glycemic control data at the end of the longitudinal phase. The percentage of T2DM patients who achieved glycemic target HbA1c <7% increased from 17.8% at baseline to 38.4% after nine months of follow-up. The glycemic goals, as targeted by the treating physician, were achieved by 77.4% at the end of the follow-up, compared to 28.3% at the initial visit (Fig. **2**).

#### 3.3.2 Change in the Prescription Pattern of Antidiabetic Medications and Lifestyle Modifications

Overall, 158 (87.8%) of the T2DM patients received OAD medications either alone (n =129) or in combination with insulin (n =29). The most commonly prescribed OADs for T2DM were metformin alone (12.8%), sulfonylureas alone (8.9%), or metformin plus sulfonylureas (36.1%). The percentage of people treated with OADs was stable at the end of follow-up (88.2%), with 7.6% of people who had their OADs prescription modified during the longitudinal period; no patients started an OADs treatment at the end of follow-up or discontinued their OADs at the end of follow-up (Table **3**).

The proportion of T2DM patients who followed a healthy diet and exercise plan increased from 39.4% at baseline to 71.2% at the end of follow-up.

#### 3.3.3 Diabetes-related Complications

Over the past year, 14.9% of the patients experienced at least one diabetes-related complication; 10.9% had at least one microvascular complication, and 6.3% had at least one macrovascular complication. During the longitudinal period, new or worsening diabetes-related complications were reported in 3.5% of people since baseline: among them, 40.0% had microvascular complications, and no patients had macrovascular complications since the baseline visit (Table **3**).

In terms of safety, 5.3% experienced symptomatic episodes of hypoglycemia in the past three months, and only one patient experienced severe episodes of hypoglycemia (requiring assistance) in the past 12 months. Hospitalizations due to diabetes and its complications were reported for three T2DM patients (2.2%) during the past 12 months. One T2DM patient experienced hypoglycemia symptoms during the longitudinal period, and no T2DM patient experienced any severe hypoglycemia episode during the longitudinal period (Table **3**).

### 3.4 Diabetes Medication Adherence and Education

**Fig. (3):**
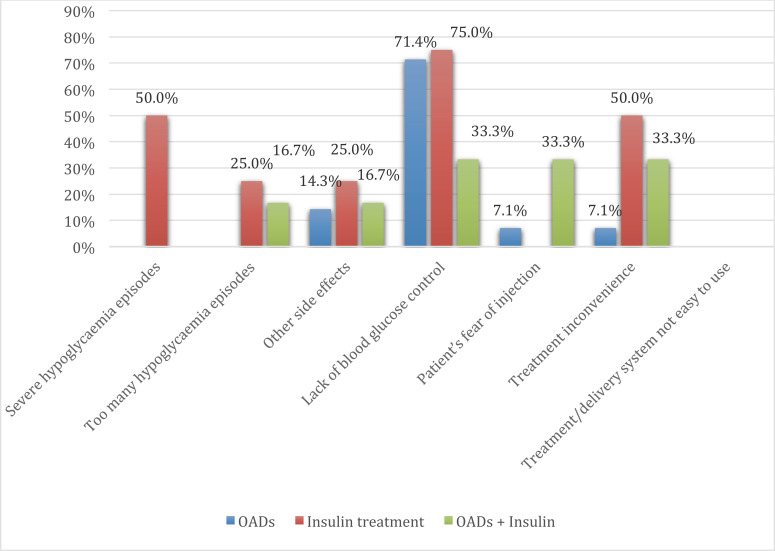
The reasons for non-adherence to diabetic medications. **Abbreviation: OADs:** Oral antidiabetic drug.

Approximately, 85.5% of T2DM patients followed their diabetes medication dosage and frequency strictly as prescribed. None of the patients on insulin therapy reported discontinuation. The reason for non-adherence was mainly due to a lack of blood glucose control (62.5%), financial consideration (62.5), and diet constraints (45.8%) (Fig. **3**). One-quarter of the patients (44 patients; 24.4%) had a glucose meter, and most of them (31 patients) self-monitored their blood glucose with the glucose meter. Overall, 38.3% of T2DM patients received diabetes education, and 28.9% were involved in an educational program provided by the physician or their clinical staff.

## 4 DISCUSSION

In this study, our findings showed that the proportion of insulin use among T2DM patients was 21.7%, with a mean duration of 32.4–36.6 months. The insulin was mainly received in combination with OADs (74.4%). This proportion is comparable with the major studies that assessed the trends in using insulin alone or combined with OADs in patients with T2DM. The US National Health and Nutrition Examination Survey (NHANES) showed a decreased trend in using insulin alone, from 26.8% between 1988 and 1994 to 14.1% between 2005 and 2012. In contrast, there was an increasing trend in the use of insulin in addition to OADs, from 3.5% between 1988 and 1994 to 14.9% between 2005 and 2012 [19]. Between 2009–2014, the US NHANES reported that the percentage of patients treated with insulin among those diagnosed with T2DM was 22.2% [20]. Similarly, the DiabCare study of the Philippines showed that in 2008, about one-quarter of the T2DM patients used insulin alone and/or in combination with OADs [21]. Another Asian study from the Joint Asia Diabetes Evaluation Registry demonstrated that 21% of T2DM patients received insulin therapy to control their diabetes [22]. In India [23], Korea [24], Japan [25], Turkey [26], and South Africa [27], the percentage was much lower at 9.1%, 3%, 7%, 9.6%, and 4.4%, respectively. In Europe, the rate of T2DM insulin users was 12.5% in the UK [28], 11.7% in Sweden [29], and 15.8% in Denmark [30]. In addition to the high percentage of uncontrolled patients in our study, these estimates revealed that the current use of insulin therapy in T2DM patients is still insufficient; thus, improving clinical practice to be more adherent to global guidelines and improving insulin accessibility are required. According to Basu and his colleagues, prescribing insulin for better glycemic control and making insulin more widely available will increase global insulin therapy use from 7.4% to 15.5% by 2030 [31].

The ADA/EASD consensus recommends insulin therapy when HbA1c is above target despite dual/triple therapy, injectable combinations, especially when HbA1c is above 10% or 2% above target [14]. Furthermore, if HbA1c is very high (> 11%) and/or there are symptoms or indications of catabolism, insulin should be considered at any time [14]. Several studies have suggested that optimal insulin therapy for T2DM may include the early initiation of a basal insulin regimen, with subsequent addition and intensification of prandial or premixed insulin in addition to basal insulin, and ultimately a full basal-bolus regime [15, 16], much earlier than currently practiced [32].

More than half of the patients (56.4%) were on basal insulin, mainly long-acting basal insulin (90.9%). In addition, 46.2% of the patients were on premixed insulin, with human-premixed insulin accounting for the vast majority of these cases (83.3%). Most patients were on premixed insulin alone (43.6%), followed by basal alone (41%). Five (12.8%) patients were on prandial insulin combined with basal analogues. Sarkar *et al.* showed that in the USA, the use of long-acting insulin accounts for 74.8% of the T2DM patients who received insulin therapy compared to 16.5% in terms of rapid-acting insulin. Premixed insulin was used in only 6%. Regarding the insulin type, analogue insulin was observed in 86.3%, while human insulin was used in 5.5% [33]. Many individuals reported that adding basal insulin to the OADs is quite effective. However, 30–50% of patients initiating insulin treatment with basal insulin did not meet the 7% HbA1c target, according to Raccah *et al.* [34]. As a result, even individuals who achieve great HbA1c control with basal insulin may need to treat postprandial hyperglycemia later. Patients with T2DM who were poorly managed on basal insulins might improve their glycaemic control by switching to premixed insulin treatment, according to the findings of the IMPROVE^TM^ subgroup study. Switching to premixed insulin, regardless of their previous basal insulin regimen, allowed many patients to achieve their HbA1c target without hypoglycemia in most cases [35]. Our findings showed an overuse of human insulin compared to other studies [33]. Many studies reported that human insulin is less effective than insulin analogues; however, its price is more affordable, explaining the overuse [36]. A meta-analysis of 13 RCTs demonstrated that short-acting insulin analogues provide better control of HbA1c and postprandial glucose in T2DM than regular human insulin, with no substantial reduction in the risk of severe hypoglycemia [37].

Notably, 28.2% of the patients received insulin *via* vials, and 46.2% stated that they adjusted the insulin dose by themselves. According to Sarkar and his team, insulin vials/syringes use in American patients with T2DM has reduced from 63.9% in 2016 to 41.1% in 2020, while insulin pens have increased from 36.1% to 58.7% between 2016 and 2020 [33]. Many studies have demonstrated that using insulin pen devices in T2DM patients who initiate insulin glargine was associated with lower HbA1c levels and better adherence than vials or syringes. Moreover, both methods have comparable costs [38, 39]. In insulin-naïve patients with T2DM, patients preferred pens over vials/syringes when initiating basal insulin treatment (P<0.001) [40]. Xie *et al.* reported that in insulin-naive patients with T2DM, initiation of insulin glargine using the disposable pen rather than the vial/syringe is associated with higher persistence, better HbA1c control, and lower rates of hypoglycemia [41].

In our study, 38.4% of the T2DM patients achieved the glycemic target (HbA1c < 7%), and 77.4% achieved the physicians’ target. None of the T2DM patients reached the triple target. These percentages highlighted the unmet needs in managing T2DM patients in Egypt, which could be attributed to the delay of insulin therapy, overuse of human insulin, overuse of vials, lack of diabetes education, and lack of healthcare support. Insulin-treated individuals who feel highly empowered by their healthcare providers may feel more in control of their diabetes, and as a result, become more involved in disease management, resulting in lower HbA1c levels, thus highlighting the role of continuous support from healthcare providers [42]. In the cross-sectional study by Chen and his colleagues, 83.5% of all insulin-treated patients with diabetes had current HbA1c levels <7%, comparable to our findings [42]. This finding is similar to that of a study by Angamo *et al.*, in which 81.7% of insulin-treated patients had HbA1c levels <7% [43]. In the Chinese population, a study found a higher proportion of adequate glycemic control in patients who received insulin-only compared with those who received OADs-only (41.8% *vs.* 35.9%; OR=1.48, 95% CI:1.09-2.01). However, patients in the insulin-only group experienced a higher rate of hypoglycemia than patients in the OADs-only (33.3% *vs.* 14.4%; OR:2.38, 95%CI: 1.72-3.29) [44]. The rate of hypoglycemia in our study was 5.3% in T2DM patients, which was better than reported in the study of Chen *et al.* [44].

Patients with T2DM are prone to microvascular, macrovascular, and neuropathic complications, which account for significant morbidity and mortality worldwide [45, 46]. The present study found that 14.9% of T2DM patients experienced at least one diabetes-related complication, mostly microvascular complications. Such findings were in line with a multinational study, “A1chieve study,” on 66726 T2DM patients from 28 countries showing a high rate of complications among diabetic populations with a higher rate for microvascular complications than macrovascular complications (53.5% and 27.2%, respectively) [47]. Another report found that approximately 38.5% of T2DM complained of one or more diabetic complications, mostly microvascular complications [48].

Patient education has a strongly useful impact on diabetic patients. The education promotes patients’ awareness regarding the nature of the disease and its complications, which, in turn, helps delay or prevent diabetic complications and improve the quality of life. Multiple education methods have been assessed and proved to have a significant impact on the patient and community levels as the reduction in the cost of health care. In light of that, a published systematic review by Duke *et al.* included a total of 1359 patients diagnosed with T2DM from nine controlled clinical trials. The included patients had an individual education. They concluded that diabetic patient education has a significant benefit on the glycemic control of the patients compared to usual care [49].

Regarding adherence, our findings showed that 85.5% of T2DM patients followed their diabetes medication dosage and frequency strictly as prescribed. These proportions aligned with those reported by Chen *et al.*, who found that complete adherence was observed in 89.8% and partial adherence in 10.2% of insulin-treated patients with T2DM [42]. However, adherence to recommended insulin regimens, in terms of frequency and dosage, was relatively high in our study compared with previous studies by Farsaei *et al.* [50] and Mashitani *et al.* [51]. When a population’s adherence rate is high, the influence of adherence behaviors on HbA1c levels is minimal [52]. This phenomenon might explain why current HbA1c levels were not substantially linked with insulin regimen adherence in the present study’s injection frequency and dose.

The social determinants of health (SDOH) include aspects, such as availability, cost, and quality of health care. The prevalence, financial implications, and disproportionate population burden of diabetes make an understanding and mitigation of SDOH a priority [53, 54].

Diabetes patients’ healthcare expenses are 2.3 times higher than non-diabetics [55]. Due to cost, around 14%-20% of individuals with diabetes report lowering or postponing treatment; rates may exceed 25% among persons with diabetes who are taking insulin [56-58]. Income, insurance coverage, and the availability of cost-sharing reduction strategies are all connected to cost-related or cost-reducing non-adherence (CRN). CRN is linked with higher levels of economic strain, financial insecurity, and economic limitations [57, 59], and it is associated with worse diabetes control, a higher HbA1c, and a worse functional status [59, 60]. Insulin CRN has been linked to deaths in both children and adults with T1DM [61].

Adults with diabetes are more likely to receive necessary tests and treatment if they have health insurance [62], and those without health insurance are more likely to have undetected diabetes [63]. Uninsured persons with diabetes are less likely to have office visits (by 60%), receive medications (by 52%), and have more ER visits (by 168%) than their insured counterparts [55]. Having insurance has also been shown to reduce the link between economic barriers and elevated HbA1c levels [59].

## CONCLUSION

In conclusion, insulin use in patients with T2DM is increasing; however, it is still inadequate to achieve targeted glycemic control. The use of vials/syringes should be reduced and replaced with new methods and pen devices. Insulin therapy should be started as soon as current guidelines suggest, and increasing therapy intensity does not ensure glycemic targets. The level of suboptimal glycemic control in Egypt is high, requiring a multidisciplinary effort to formulate effective health policies. Nearly one-third of Egyptian patients received diabetes education; nonetheless, the adherence level is high.

## Figures and Tables

**Table 1:** Demographic and Clinical Characteristics of the Included Patients.

**Variable**	**T2DM (N =180)**
**Age (years), Mean (SD)**	53.6 (11.0)
**Male, No (%)**	78 (43.3%)
**Urban area, No (%)**	166 (92.2%)
**University/Higher education, No. (%)**	103 (57.2%)
**Type of health insurance, No. (%)**	
- No	93 (51.7%)
- Public	58 (32.2%)
- Private	18 (10%)
- Public + Private	11 (6.1%)
**Smoking habits, No. (%)**	
- Never	138 (76.7%)
- Former	17 (9.4%)
- Current	25 (13.9%)
**Duration of diabetes (years), Mean (SD)**	8.8 (7.4)
**Family history of DM, No. (%)**	106 (66.3%)
**Last HbA1c (%), Mean (SD)**	8.36 (1.72)
**HbA1c >7%, No. (%)**	138 (82.6%)
**Last FBP (mg/dL), Mean (SD)**	165.33 (58.29)
**Last PPBG (mg/dL), Mean (SD)**	216.71 (71.88)
**BMI (kg/m^2^), Mean (SD)**	31.07 (5.81)
**SBP (mmHg), Mean (SD)**	133.8 (14.6)
**DBP (mmHg), Mean (SD)**	82.4 (8.3)
**Patient diagnosed with hypertension, No (%)**	81 (47.1%)
**Patient treated for hypertension, No (%)**	N= 81 76 (93.8%)
**LDL (mg/dL), Mean (SD)**	122.51 (44.19)
**Total cholesterol (mg/dL), Mean (SD)**	207.86 (48.99)
**Dyslipidemia, No. (%)**	42 (26.1%)
**Serum Creatinine (mg/dl), Mean (SD)**	0.97 (0.35)
**eGFR (mL/min/1.73 m^2^), Mean (SD)**	78.62 (25.60)
**CKD, No. (%)**	N = 101
- Stage 1	33 (32.7%)
- Stage 2	39 (38.6%)
- Stage 3	27 (26.7%)
- Stage 4	1 (1%)
- Stage 5	1 (1%)

**Abbreviations: T2DM:** Type 2 diabetes mellitus; **SD:** Standard deviation; **FBG:** fasting blood glucose; **PPBG:** Postprandial blood glucose; **DM:** Diabetes mellitus; **BMI:** Body mass index: **SBP:** Systolic blood pressure; **DBP:** Diastolic blood pressure.

**Table 2:** The characteristics of insulin therapy in patients with T2DM.

Variables	Total (n =39)	
	**No.**	**%**
**Duration of insulin treatment (months)**	32.4 (36.6)	
**Insulin treatment started >= 3 months before the visit**	28	75.70%
**Basal insulin**	22	56.40%
Type of basal insulin*		
Long-acting insulin analogue	20	90.90%
Intermediate human insulin	0	
Biosimilar insulin	2	9.10%
**Basal insulin daily dose (IU)**	21.8 (10.8)	
**Basal insulin daily dose (IU/kg)**	0.3 (0.1)	
**Basal insulin number of injections**	1.1 (0.4)	
**Prandial insulin**	5	12.80%
Type of prandial insulin*		
Short-acting insulin analogue	4	80.00%
Rapid-acting human insulin	0	
Biosimilar insulin	1	20.00%
**Prandial insulin daily dose (IU)**	19.0 (8.2)	
**Prandial insulin daily dose (IU/kg)**	0.2 (0.0)	
**Prandial insulin number of injections**	2.4 (0.9)	
**Premix insulin**	18	46.20%
Type of Premix insulin*		
Premixed analogue insulin	3	16.70%
Premixed human insulin	15	83.30%
**% of basal / % of prandial**		
25/75	0	
30/70	5	27.80%
50/50	2	11.10%
60/40	0	
65/35	0	
70/30	11	61.10%
75/25	0	
**Premix insulin daily dose (IU)**	55.7 (18.2)	
**Premix insulin daily dose (IU/kg)**	0.7 (0.3)	
**Premix insulin number of injections**	1.9 (0.3)	
Devices used by the patient**		
Reusable pen	15	38.50%
Disposable pen	16	41.00%
Vials	11	28.20%
Pump	0	
**Patient self-adjusts insulin dose**	18	46.20%

**Note:** *A patient could receive more than one year of basal/prandial/premix insulin, **A patient could use more than one device.

**Table 3:** Management of Care of the Included Patients at CS and L Phases.

**Variable**	**CS Phase** **(N =180)**
**Type of OADs, No. (%)**	
- **No OADs**	22 (12.2%)
- **Metformin alone**	23 (12.8%)
- **Sulphonylureas alone**	16 (8.9%)
- **Metformin + Sulphonylureas (+/- others)**	65 (36.1%)
- **Other**	54 (30%)
**Number of OADs, No. (%)**	
- **1 OAD**	46 (29.1%)
- **2 OADs**	92 (58.2%)
- **More than 2 OADs**	20 (12.7%)
**GLP-1 analogues, No. (%)**	5 (2.8%)
**Diet and lifestyle modification, No. (%)**	67 (37.2%)
**Any diabetes-related complication**	26 (14.9%)
**At least one microvascular complication**	19 (10.9%)
**At least one macrovascular complication**	11 (6.3%)
**The patient experienced any symptomatic episodes of hypoglycemia in the past 3 months**	9 (5.3%)
**The patient experienced any severe episodes of hypoglycemia (requiring assistance) in the past 12 months**	1 (0.6%)
**Hospitalization due to diabetes and its complications during the past 12 months**	3 (2.2%)
**The patient received diabetes education, No. (%)**	69 (38.3%)
**The patient involved in any educational program, No. (%)**	52 (28.9%)
**Setting where the education was delivered, No. (%)**	
- **Clinic attended by the patient**	64 (92.8%)
- **Hospital-based diabetes center**	6 (8.7%)
- **Community-based diabetes center**	4 (5.8%)
- **Patient support group**	2 (2.9%)

## Data Availability

The data and supportive information are available within the article.

## LIST OF ABBREVIATIONS

T2DM: Type 2 Diabetes Mellitus
DM: Diabetes Mellitus
IDF: International Diabetes Federation
OHAs: Oral hypoglycemic Agents
HbA1c: Hemoglobin A1c
UKPDS: UK Prospective Diabetes Study
IDMPS: International Diabetes Management Practices Study
CRF: Case Report Form
SD: Standard Deviation
